# Levothyroxine dosages during pregnancy among hypothyroid women. An experience from a tertiary care center of Karachi, Pakistan, based on data from Maternal Hypothyroidism on Pregnancy Outcomes Study (MHPO-5)

**DOI:** 10.1186/s13104-022-05984-7

**Published:** 2022-03-07

**Authors:** Zareen Kiran, Wardah Khalid, Aisha Sheikh, Najmul Islam

**Affiliations:** 1 Section of Endocrinology, Department of Medicine, Aga Khan University Hospital, Stadium Road, Karachi, Pakistan; 2 University of Edinburgh, Edinburgh, UK; 3 Section of Endocrinology, Department of Medicine, Aga Khan University Hospital, Karachi, Pakistan; 4 Section of Endocrinology, Department of Medicine, Aga Khan University Hospital, Karachi, Pakistan

**Keywords:** Levothyroxine, Dosage, Thyroid, Subclinical, Overt, Pregnancy

## Abstract

**Objectives:**

The dosage of levothyroxine (LT4) during pregnancy differs among different ethnic groups worldwide. These differences are due to variations in geographical iodine distribution, autoimmunity, and variations in thyrotropin (TSH) targets for pregnancy. To the best of our knowledge, we report the levothyroxine dosage prescribed during pregnancy in hypothyroid women, for the first time from Pakistan.

**Results:**

Levothyroxine dosage of 280 hypothyroid women during pregnancy were reviewed. The median LT4 dosages prescribed before conception was 85.7 mcg per day which increased by 14.3 mcg per day in the first trimester (P 0.001). A significant difference in dosage was observed between controlled and uncontrolled TSH groups in the first trimester (P 0.05). Lower LT4 dosage was prescribed for subclinical hypothyroid women as compared to overt hypothyroid cases, whereas dosages did not differ according to autoimmune status in the latter part of gestation.

**Supplementary Information:**

The online version contains supplementary material available at 10.1186/s13104-022-05984-7.

## Introduction

Pregnancy poses many challenges in managing hypothyroidism due to several reasons. A woman’s physiological demand for thyroid hormone (T4) increases during pregnancy, which leads to incremental dosage of prescribed T4 to maintained euthyroid state [1, 2]. The daily iodine intake of 250 mcg is recommended in pregnancy, but this is not always achieved even in iodine-sufficient parts of the world [3]. Additionally, not only the urinary iodine losses are increased in pregnancy, but the degradative metabolism of tetraiodothyronine is also accelerated.

Management of hypothyroidism in pregnancy is also an added burden on the health care setup, especially in a developing country like Pakistan [4]. In 2013, a study from a tertiary care center in Lahore, reported increased levels of thyrotropin stimulating hormone (TSH) throughout pregnancy in more than 2% of women, which suggests decreased thyroid reserve during pregnancy [5]. Another multicentre study reported higher TSH levels in 4.44% of women diagnosed with overt as well as subclinical hypothyroidism, this probably suggests inadequate replacement during pregnancy [6]. Adverse effects on obstetric and neonatal outcomes like preterm birth, and postpartum hemorrhage, due to uncontrolled hypothyroidism during pregnancy has clear evidence from scientific literature [7–9]. Therefore, most evidence-based guidelines recommend adequate replacement of thyroid hormone not only during pregnancy but even before conception to reduce adverse outcomes [1, 10–12].

Levothyroxine (LT4) remains the mainstay of treatment of hypothyroidism in pregnant women worldwide [13, 14]. Women already diagnosed with hypothyroidism should have their TSH levels checked before conception or as soon as their pregnancy is confirmed to timely adjust the dosage of LT4 [15]. Various studies have proposed, that hypothyroid women can increase their LT4 dosage by approximately 30% as soon as the pregnancy is confirmed [16, 17]. Various guidelines also recommend the adjustment of LT4 dosage in hypothyroid pregnant women, but evidence is inconclusive about the time and change in the dosage required. According to the American thyroid association (ATA) guidelines TSH levels should be kept ≤ 2.5 mIU/mL throughout the pregnancy [10]. On the other hand, the Endocrine Society guideline suggests TSH remains ≤ 2.5 mIU/mL in the first trimester and up to 3 mIU/mL in second and third trimesters or in the trimester-specific TSH ranges [11]. Besides, the dose requirements for different etiologies like autoimmune versus nonautoimmune hypothyroidism are also not well established [18]. Moreover, there is no consensus on the LT4 dosage requirement in subclinical and overt hypothyroidism [7, 13, 19].

There is a lack of data and guidelines to support the recommended range of TSH and LT4 dosage to be maintained during pregnancy for the Pakistani population. To the best of our knowledge, levothyroxine dosage prescribed and its trend during pregnancy is never reported from Pakistan. Therefore, we aimed to report the prescribed LT4 dosage and the change in dose from preconception period throughout gestation in hypothyroid women from a tertiary care center. We also studied the relationship between TSH levels and LT4 dosage during each trimester and reported dosage between subclinical versus overt hypothyroid and autoimmune versus nonautoimmune hypothyroid groups.

## Main text

### Methods

We conducted a retrospective chart review of hypothyroid pregnant patients presenting at the endocrine and obstetric clinics of Aga Khan University Hospital (AKUH), Karachi, Pakistan. AKUH is a Joint Commission International (JCI)-accredited tertiary health care center where people from all over the country are visiting for specialist care. It has well-established obstetrics and gynecological department which works in close collaboration with the endocrine section. Thyroid disorders in pregnancy are managed according to internationally recognized guidelines [10, 11]. All hypothyroid pregnant females presenting to AKUH from 2008 till 2016 were included in this retrospective study. The in-depth methods and other details of the whole population characteristics can be reviewed in our previously published papers [9, 20]. We reached a sample size of 280 hypothyroid pregnant women for this study objectives by applying the following eligibility criteria:

- Pregnant women of 18 years of age above, who had been diagnosed hypothyroidism, either overt (TSH > 10 mIU/mL and FT4 below normal range [10.30–23.17 pmol/L]) or subclinical (TSH > 10 mIU/mL and FT4 normal), by the clinician before pregnancy, as per chart review.
- These women were on levothyroxine replacement with varying dosages prescribed throughout pregnancy [11].

Pregnant women diagnosed with hypothyroidism during pregnancy, and those cases with incomplete levothyroxine dosages data during gestation were excluded from the study. These eligible females were identified through the international classification of diseases (ICD-10) coding system by applying coding words of hypothyroidism and pregnancy in the electronic medical record of our hospital. Data were extracted by trained medical doctors and double-checked by the principal investigator.

#### Levothyroxine dosages

Dosage of levothyroxine in the preconception period and each month throughout gestation was noted. Levothyroxine is mostly available in the form of 50 mcg tablet in our settings, so the dosage requirement per day is prescribed as a total weekly dose, distributed into a varying number of tablets per day throughout the week considering the half-life being 7 days [21]. Therefore, to calculate the LT4 dose prescribed for each month during pregnancy, we first calculated the average LT4 dosage per day from the number of tablets prescribed, then we calculated the medians of each trimester from the dosages of respective months.

#### Thyroid stimulating hormone levels

TSH levels available before conception and during each month throughout pregnancy were noted. TSH levels were assessed by Advia Centaur (Siemens Diagnostics), Chemiluminescence immunoassay. Specificity of this third generation assay was determined against hCG (human chorionic gonadotropin), FSH (follicle-stimulating hormone) & LH (luteinizing hormone) with no significant cross-reactivity observed. The functional sensitivity of the assay is 0.008 mIU/mL, with the reference range from 0.008–150 mIU/mL [22, 23]. We do not have trimester specific ranges available in our institute, therefore we follow non pregnant adult TSH ranges being used in our hospital, that is, 0.4–4.2 mIU/mL, throughout pregnancy [24].

Statistical analysis was performed using the software of Statistical Package for the Social Sciences version 22.0. Baseline clinical features of hypothyroid pregnant women were reported as Mean ± SD for normally distributed continuous data and as Median with interquartile range (IQR) for skewed distributed data. For categorical variables, frequencies with percentages were reported. The LT4 dosage was reported as median (IQR) for each trimester along with the preconception period. Medians (IQR) of LT4 dosage were also determined for subclinical and overt hypothyroid groups as well as autoimmune and nonautoimmune hypothyroid groups of pregnant women. Medians of TSH levels were also calculated and were grouped into controlled (target TSH ≤ 2.5 mIU/mL) and uncontrolled (target TSH ≥ 2.5 mIU/mL) levels**.** Differences in LT4 dosages were assessed between these two groups using the Mann–Whitney U test. Change in LT4 dose from preconception to the first trimester and through different trimesters was calculated using the Wilcoxon Signed-Rank test. A P-value of ≤ 0.05 was considered significant.

### Results

A total of 718 hypothyroid cases were identified through ICD-10 coding. Out of these 718 cases, 10 cases were excluded due to missing data for the complete course of pregnancy. From the rest of the 708 cases, those with missing LT4 dosages in the preconception period were excluded. Then cases with missing LT4 dosages in each trimester were excluded, finally selecting 280 participants (Additional File 1: Figure S1). The mean age of the study participants was 30.9 (4.62) years. Majority of these women presented in the 6th week of the first trimester (69%). Up to 37.5% were subclinical and 18.2% had overt hypothyroidism, while the rest of the cases were unclassified. Only 32% of women had their antibodies checked. Thyroid peroxidase antibodies were positive in 19.6% and anti-thyroglobulin was positive in 16.8% cases. Diabetes mellitus (DM) was present in 6.8%, 20% had gestational DM, 4.3% had chronic hypertension and 11.5% had gestational hypertension. Positive family history of hypothyroidism was present for 20.4% of participants (13.9% in first-degree and 5% in second-degree relatives). Around 99% had a live birth and only 1 had intrauterine death.

#### TSH levels and the Levothyroxine dosage in each trimester

TSH levels were determined in 75.4%, 54.6%, 63.6%, and 76.4% of cases in preconception, 1^st^, 2^nd^, and 3^rd^ trimester periods respectively. Median TSH level before conception was 2.6 (3.4) mIU/mL whereas, during first, second and third trimester, it was 2.9 (4.5), 2.5 (2.2) and 2.3 (2.1) mIU/mL respectively.

Amongst 280 women, the median (IQR) dosage of levothyroxine before pregnancy was 85.7 (55.3) mcg per day (Table 1.) This was significantly lower than that at the beginning of the first trimester (100.0 mcg per day, P-value 0.001), which remained the same up to third trimester (100.0 mcg per day, P-value 0.000). The median difference in dosage was 14.3 mcg per day which translates into a 16.7 percent increase from preconception to the first trimester.

**Table 1:** Preconception and trimester-wise Levothyroxine dosage administered in hypothyroid women

Levothyroxine dosage (µg per day) n = 280 Median (IQR)		P value*
Preconception dose 85.7 (55.3)	1^st^ trimester dose 100.0 (69.6)	0.001
1^st^ trimester dose 100.0 (69.6)	2^nd^ trimester dose 100.0 (75.0)	< 0.01
2^nd^ trimester dose 100.0 (75.0)	3^rd^ trimester dose 100.0 (96.4)	< 0.01

^*^P-Value calculated by Wilcoxon Signed Rank test

Table 2 shows the differences in dosages between patients achieving controlled levels of TSH at ≤ 2.5 mIU/mL and those with uncontrolled TSH ≥ 2.5 mIU/mL. During the preconception period, there was no statistically significant difference between the 2 groups, although the controlled TSH group had a slightly higher dosage prescribed. The levothyroxine dose difference was marginally significant in the first trimester (P-value 0.05), whereas there was no difference in other trimesters.

**Table 2:** Difference of prescribed levothyroxine dosage among TSH groups according to preconception and trimester-wise status in hypothyroid women

	Levothyroxine dosage in controlled TSH levels (≤ 2.5 uIU/mL) Median (IQR)	Levothyroxine dosage in uncontrolled TSH Levels (≥ 2.5 uIU/mL) Median (IQR)	P-value*
Preconception (n = 211)	85.7 (58.9) n = 106	75.0 (50.0) n = 105	0.20
First trimester (n = 153)	100.0 (72.3) n = 70	71.4 (64.3) n = 83	0.05
Second trimester (n = 178)	100.0 (75.8) n = 90	100.0 (64.3) n = 88	0.09
Third trimester (n = 214)	100.0 (100.0) n = 117	100.0 (71.4) n = 97	0.42

^*^P-Value calculated by Mann Whitney Test

#### Levothyroxine dosage in subclinical versus overt and autoimmune versus nonautoimmune hypothyroid pregnant women

Overall, there was a lower levothyroxine dosage prescribed in subclinical hypothyroid pregnant women (< 100 mcg per day) throughout pregnancy. On the other hand, there was a lower median dosage prescribed in the nonautoimmune group in the preconception period as compared to the autoimmune cases, although the dosage remained the same throughout each trimester except in the third trimester (Table 3).

**Table 3:** Levothyroxine dosage prescribed in subclinical and overt hypothyroid subgroups as well as autoimmune and nonautoimmune subgroups according to trimester wise and preconception status

	Subclinical hypothyroidism (N = 105) (µg/day)	Overt hypothyroidism (N = 50) (µg/day)	Autoimmune hypothyroidism (N = 78) (µg/day)	Nonautoimmune hypothyroidism (N = 202) (µg/day)
Preconception dosage*	50.0 (50.0)	100.0 (41.8)	85.7 (66.0)	75.0 (50.0)
1^st^ trimester dosage	64.3 (50.0)	100.0 (41.0)	100.0 (75.0)	100.0 (64.3)
2^nd^ trimester dosage	71.4 (50.0)	100.0 (50.0)	100.0 (87.5)	100.0 (71.4)
3^rd^ trimester dosage	78.5 (71.4)	110.7 (51.7)	103.5 (92.8)	100.0 (85.7)

^*^Dosage reported as medians (IQR)

## Discussion

Our study reported a 16.7% increase in levothyroxine dosage prescribed from preconception to the first trimester. This is lower than around 30–50% increment reported in the literature [16, 25]. This is also lower than the recommended increment in dosage at first confirmation of pregnancy, by international guidelines [10, 11]. In our study, this was enough for the TSH target of ≤ 2.5 mIU/mL, which was achieved in the early period of gestation in the group with higher dosage in our study (Table 2). However, thereafter the dosages were similarly prescribed throughout the remaining gestation, regardless of whether the target TSH levels were achieved or not.

Several studies have reported this plateau effect of dosage prescription in the latter part of gestation [26]. Although this pattern of dosage requirement can be the result of the changes in thyroid pathophysiology during early pregnancy, the effect on the rest of the gestation cannot be explained. Whether having more subclinical cases and majority with nonautoimmune etiology is the reason for this observation, needs to be analyzed through prospective studies.

There was a lower dosage prescribed in the subgroup of subclinical hypothyroidism patients as compared to overt hypothyroidism in our cohort. This is well explained by the fact that some of the thyroid reserves remain functional enough to compensate for the increased thyroid hormone requirement in pregnancy [27]. However, with the advancement in gestation, the LT4 dosage prescribed continues to increase in this group whereas it remains the same in the overt hypothyroid group, suggesting ongoing stress on the thyroid gland [27]. Similarly, women with autoimmune pathophysiology had higher early pregnancy dosage prescribed as compared to nonautoimmune hypothyroid women. This increased dosage in autoimmune hypothyroidism during pregnancy has clear reported evidence [27, 28], but some studies suggest no difference in dosages concerning the etiology of hypothyroidism [18].

### Conclusion

Our study concludes, a median difference of 14.3 mcg/day dosage in LT4 in the first trimester from preconception. Further outcome studies are required to understand the implication of this difference on our population and whether our TSH target level during pregnancy needs to be redefined.

## Limitations

Our study has several limitations. We cannot assess the response in TSH levels as the dose of LT4 was adjusted over time due to lack of complete data of TSH levels. The study participants in subgroup analysis were also not equally distributed. However, to the best of our knowledge, despite being a retrospective study, it is the first to report about the LT4 dosage pattern prescribed in hypothyroid pregnant women from Pakistan. We recommend large prospective studies to assess the LT4 dosages requirements in hypothyroid pregnant women from our region.

## Supplementary Information

Additional file 1:** Figure. S1 **Schematic diagram of final number of hypothyroid pregnant women selected as study participants

## Data Availability

The datasets used and/or analyzed during the current study are available from the corresponding author on reasonable request. Corresponding author’s email is drzareenkiran@gmail.com.

## Abbreviations

T4: Thyroid hormone
TSH: Thyrotropin stimulating hormone
LT4: Levothyroxine
hCG: Human chorionic gonadotropin
FSH: Follicle-stimulating hormone
LH: Luteinizing hormone
ATA: American thyroid association
JCI: Joint Commission International
AKUH: Aga Khan University Hospital
ICD-10: International classification of diseases-10
QR: Interquartile range
DM: Diabetes mellitus
