# Supplementary material for: Levothyroxine dosages during pregnancy among hypothyroid women. An experience from a tertiary care center of Karachi, Pakistan, based on data from Maternal Hypothyroidism on Pregnancy Outcomes Study (MHPO-5)
Source: BMC Res Notes. 2022 Mar 7;15:92. doi: 10.1186/s13104-022-05984-7 (PMC8900377; doi:10.1186/s13104-022-05984-7)
Supplement: Supplementary file 1 — Additional file 1: Figure. S1 Schematic diagram of final number of hypothyroid pregnant women selected as study participants [file 13104_2022_5984_MOESM1_ESM.docx]

**Figure S1. Schematic diagram of final number of hypothyroid pregnant women selected as study participants.**
